# Ru(II)-thymine complex causes DNA damage and apoptotic cell death in human colon carcinoma HCT116 cells mediated by JNK/p38/ERK1/2 via a p53-independent signaling

**DOI:** 10.1038/s41598-019-47539-0

**Published:** 2019-07-31

**Authors:** Suellen L. R. Silva, Ingrid R. S. Baliza, Rosane B. Dias, Caroline B. S. Sales, Clarissa A. Gurgel Rocha, Milena B. P. Soares, Rodrigo S. Correa, Alzir A. Batista, Daniel P. Bezerra

**Affiliations:** 1 Gonçalo Moniz Institute, Oswaldo Cruz Foundation (IGM-FIOCRUZ/BA), Salvador, Bahia 40296-710 Brazil; 2 Department of Biomorphology, Institute of Health Sciences, Federal University of Bahia, Salvador, Bahia 40110-902 Brazil; 3 Department of Chemistry, Federal University of Ouro Preto, Ouro Preto, Minas Gerais 35400-000 Brazil; 4 Department of Chemistry, Federal University of São Carlos, São Carlos, São Paulo 13561-901 Brazil

**Keywords:** Pharmacology, Drug development

## Abstract

Ru(II)-thymine complex [Ru(PPh_3_)_2_(Thy)(bipy)]PF_6_ (where PPh_3_ = triphenylphosphine, Thy = thyminate and bipy = 2,2′-bipyridine) is a potent cytotoxic agent with ability to bind to DNA, inducing caspase-mediated apoptosis in leukemia cells. In this study, we investigated the mechanism underlying the cell death induction by Ru(II)-thymine complex in human colon carcinoma HCT116 cells, as well as its effect in xenograft tumor model. The Ru(II)-thymine complex increased significantly the percentage of apoptotic HCT116 cells. Co-treatment with a JNK/SAPK inhibitor, p38 MAPK inhibitor and MEK inhibitor, which inhibit the activation of ERK1/2, caused a marked reduction of the percentage of complex-induced apoptotic cells. Moreover, the Ru(II)-thymine complex induced an increase in phospho-JNK2 (T183/Y185), phospho-p38α (T180/Y182) and phospho-ERK1 (T202/Y204) levels in HCT116 cells. Treatment with the Ru(II)-thymine complex increased significantly the phospho-histone H2AX (S139) expression, a DNA damage marker. The expression of phospho-p53 (S15) and MDM2 were not changed, and the co-treatment with a p53 inhibitor (cyclic pifithrin-α) did not reduce the complex-induced apoptosis in HCT116 cells, indicating that the Ru(II)-thymine complex induces DNA damage-mediated apoptosis by JNK/p38/ERK1/2 via a p53-independent signaling. The Ru(II)-thymine complex (1 and 2 mg/kg/day) also inhibited HCT116 cell growth in a xenograft model, reducing the tumor mass at 32.6–40.1%. Altogether, indicate that the Ru(II)-thymine complex is a promising anti-colon cancer drug candidate.

## Introduction

Colorectal cancer is the third highest incidence of cancer in the world and the second highest mortality. In 2018, 18.1 million new cases and 9.6 million cancer deaths were estimated worldwide, which include 1.8 million new colorectal cancer cases and 881,000 deaths^1^. The platinum-based drug oxaliplatin, along with a fluoropyrimidine chemotherapy, are among the standard colorectal cancer treatment regimens. However, drug resistance and severe side effect are some limitations of the treatment^2,3^.

Ruthenium-based antineoplastic drugs candidates are coordination complexes of ruthenium that have been reported as alternatives to platinum-based anticancer drugs, due to their reduced side effects, which may be attributed, at least in part, to the ability of this metal to mimic iron in binding to several biological molecules^4,5^. Although no ruthenium-based drug has been commercialized yet, phase I/II clinical trials with the ruthenium complexes [ImH]trans-[RuCl_4_(Im)(dmso-S)] (NAMI-A, where Im = imidazole) and [IndH]trans-[RuCl_4_(Ind)_2_] (KP1019, where Ind = indazole) have been completed^6–9^. Moreover, numerous ruthenium-based agents are currently in pre-clinical cancer drug development stage, targeting DNA interaction, cell cycle arrest, tumor angiogenesis, endoplasmic reticulum stress, reactive oxygen species induction and apoptosis activation^10–15^.

**Figure 1:**
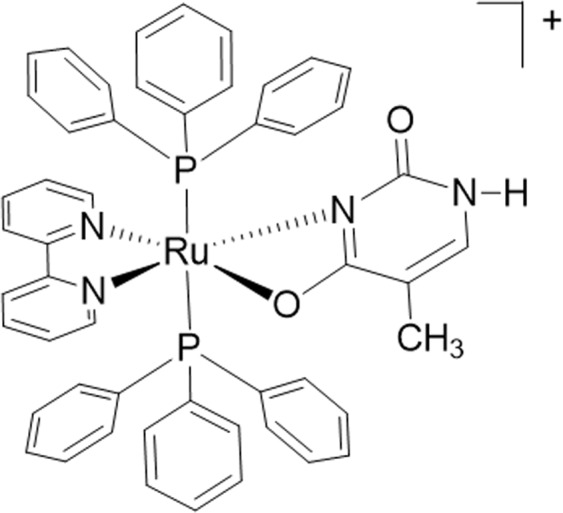
Chemical structure of the Ru(II)-thymine complex.

Ru(II)-thymine complex [Ru(PPh_3_)_2_(Thy)(bipy)]PF_6_ (where PPh_3_ = triphenylphosphine, Thy = thyminate and bipy = 2,2′-bipyridine) (Fig. 1) was previously described as a potent cytotoxic agent with the ability to bind DNA and human and bovine serum albumins, which induces caspase-mediated apoptosis in leukemia cells^16,17^. Therefore, in this present study, we investigated the cytotoxic mechanisms underlying cell death of human colon carcinoma HCT116 by the Ru(II)-thymine complex, as well as its effect in xenograft tumor model.

## Results

### Ru(II)-thymine complex causes DNA damage-mediated apoptotic cell death in HCT116 cells mediated by JNK/p38/ERK1/2 via a p53-independent signaling

**Figure 2:**
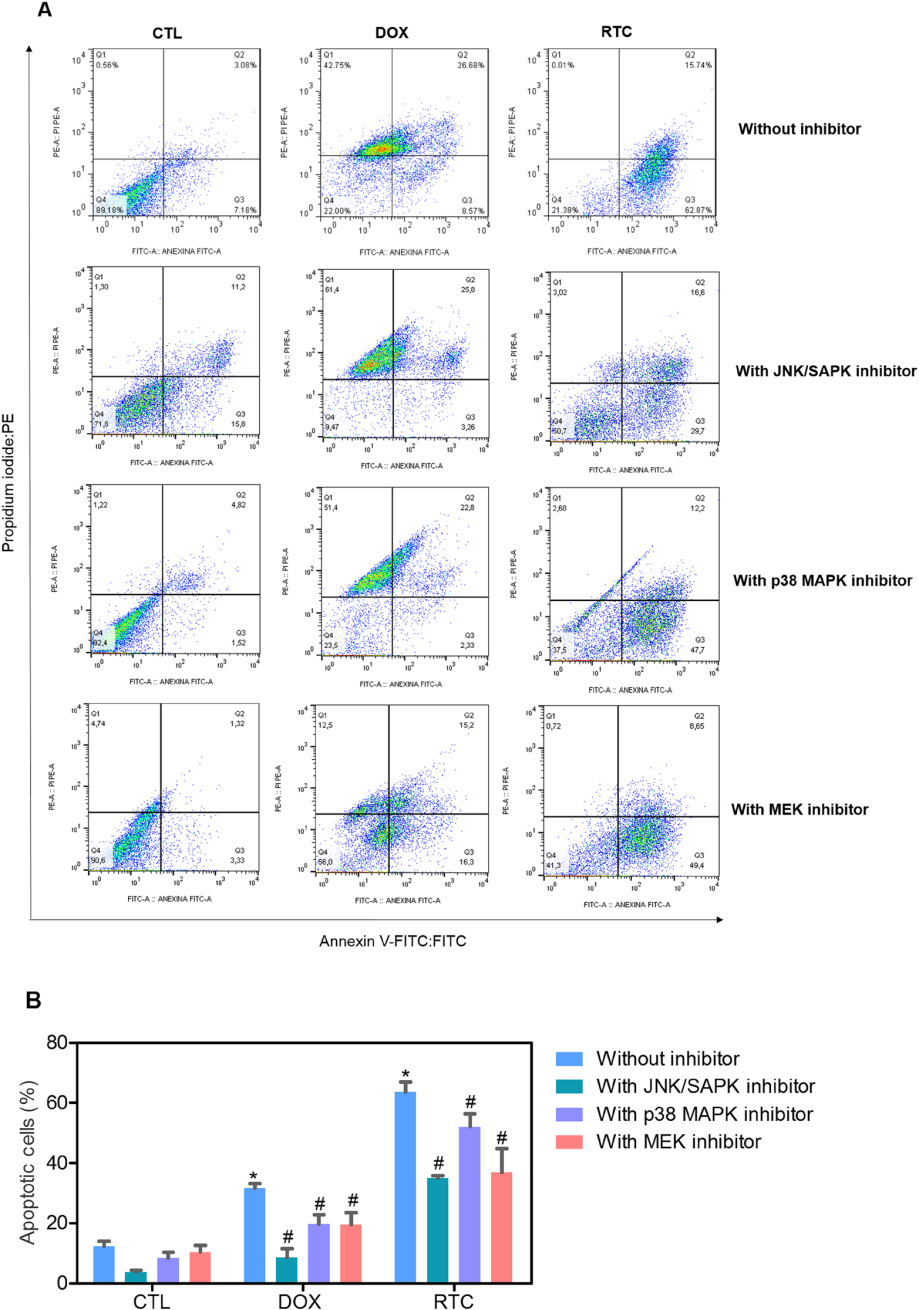
Effect of the Ru(II)-thymine complex (RTC) in the cell death of HCT116 cells with and without pretreatment with MAPK inhibitors, as determined by flow cytometry using Annexin V-FITC/PI staining. (**A**) Representative flow cytometric dot plots showing the percentage of cells in viable (annexin V-FITC negative and PI negative cells), early apoptotic (annexin V-FITC positive, but PI negative cells), late apoptotic (annexin V-FITC positive and PI positive cells) and necrotic stages (PI positive, but annexin V-FITC negative cells). (**B**) Quantification of apoptotic HCT116 cells (annexin V-FITC positive cells). The compound SP 600125 was used at 5 µM as JNK/SAPK inhibitor, PD 169316 was used at 5 µM as p38 MAPK inhibitor and U-0126 was used at 5 µM as MEK inhibitor. The cells were pretreated for 2 h with the inhibitors and then co-incubated with the complex at 4 µM for more 48 h. The negative control was treated with the vehicle (0.1% DMSO) used for diluting the complex, and doxorubicin (1 µM) was used as the positive control. Data are presented as the means ± S.E.M. of at the least three independent experiments performed in duplicate. At least 1 × 10^4^ events were recorded per sample and cellular debris was omitted from the analysis. *P < 0.05 compared with the negative control by ANOVA, followed by the Student-Newman-Keuls test. ^#^*P* < 0.05 compared with the respective treatment without inhibitor by ANOVA, followed by the Student-Newman-Keuls test.

**Figure 3:**
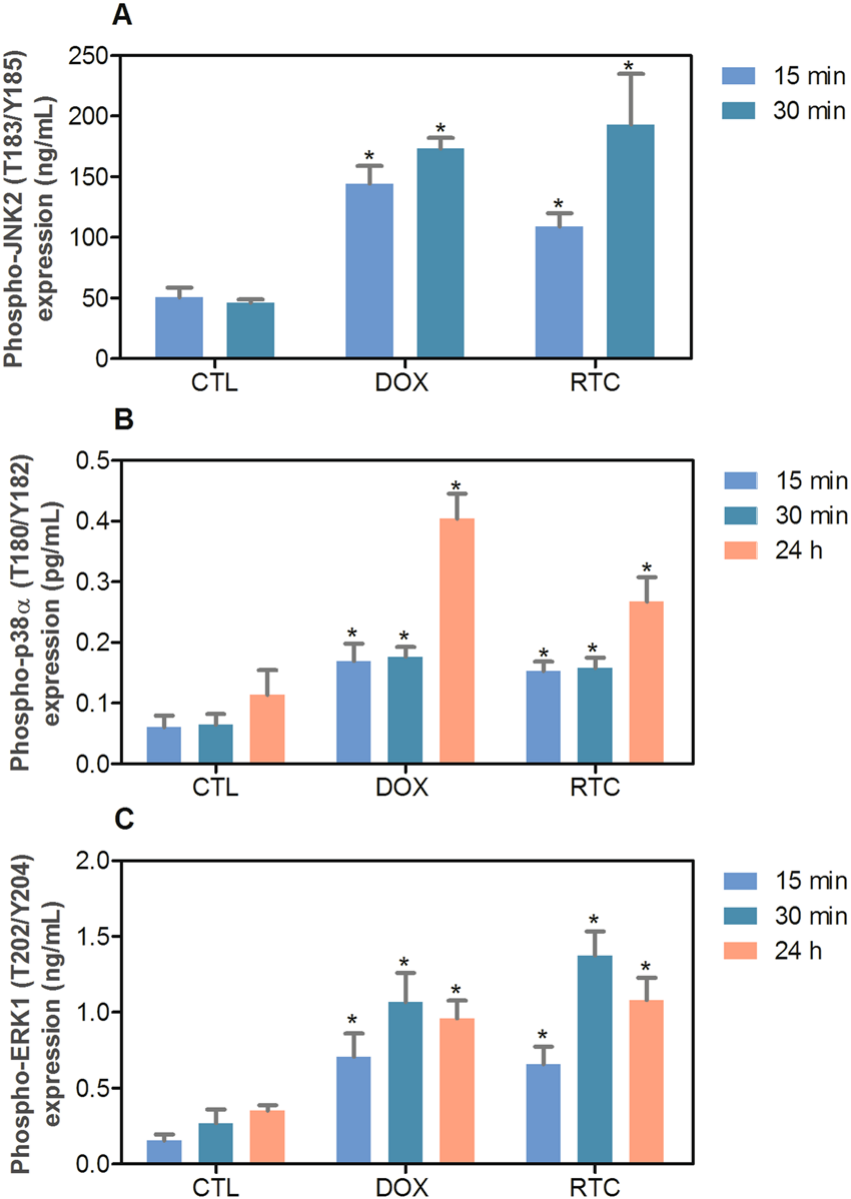
Effect of the Ru(II)-thymine complex (RTC) in phospho-JNK2 (T183/Y185), phospho-p38α (T180/Y182) and phospho-ERK1 (T202/Y204) expressions, as determined by phospho-specific ELISA in HCT116 cells. (**A**) Quantification of phospho-JNK2 (T183/Y185) expression. (**B**) Quantification of phospho-p38α (T180/Y182) expression. (**C**) Quantification of phospho-ERK1 (T202/Y204) expression. The cells were treated with the complex at 4 µM for an acute (15 or 30 min) and/or prolonged (24 h) incubation. The negative control was treated with the vehicle (0.1% DMSO) used for diluting the complex, and doxorubicin (1 µM) was used as the positive control. Data are presented as the means ± S.E.M. of at the least three independent experiments performed in duplicate. *P < 0.05 compared with the negative control by ANOVA, followed by the Student-Newman-Keuls test.

Annexin-V/PI double staining using flow cytometry was used to measure the cell death in HCT116 cells after treatment with Ru(II)-thymine complex at 4 µM after 48 h treatment. The complex significantly increased the percentage of apoptotic HCT116 cells (p < 0.05) (Fig. 2). In addition, the role of the mitogen-activated protein kinase (MAPK) pathway was assessed in the apoptotic cell death caused by this Ru(II) complex. For this, we quantified the complex-induced apoptotic HCT116 cells co-treated with a Jun N-terminal kinase/stress activated protein kinase (JNK/SAPK) inhibitor (SP600125), p38 MAPK inhibitor (PD169316) and MEK inhibitor (U-0126, which inhibits the activation of extracellular signal-regulated kinase 1 and 2 - ERK1/2). The co-treatment with JNK/SAPK, p38 MAPK and MEK inhibitors reduced significantly the percentage of complex-induced apoptotic HCT116 cells (Fig. 2). We also quantified the phospho-JNK2 (T183/Y185), phospho-p38α (T180/Y182) and phospho-ERK1 (T202/Y204) expressions in HCT116 cells treated with the complex at 4 µM after an acute (15 or 30 min) and/or prolonged (24 h) treatment. A significant increase in the phospho-JNK2 (T183/Y185), phospho-p38α (T180/Y182) and phospho-ERK1 (T202/Y204) expressions was observed in the complex-treated HCT116 cells (Fig. 3).

**Figure 4:**
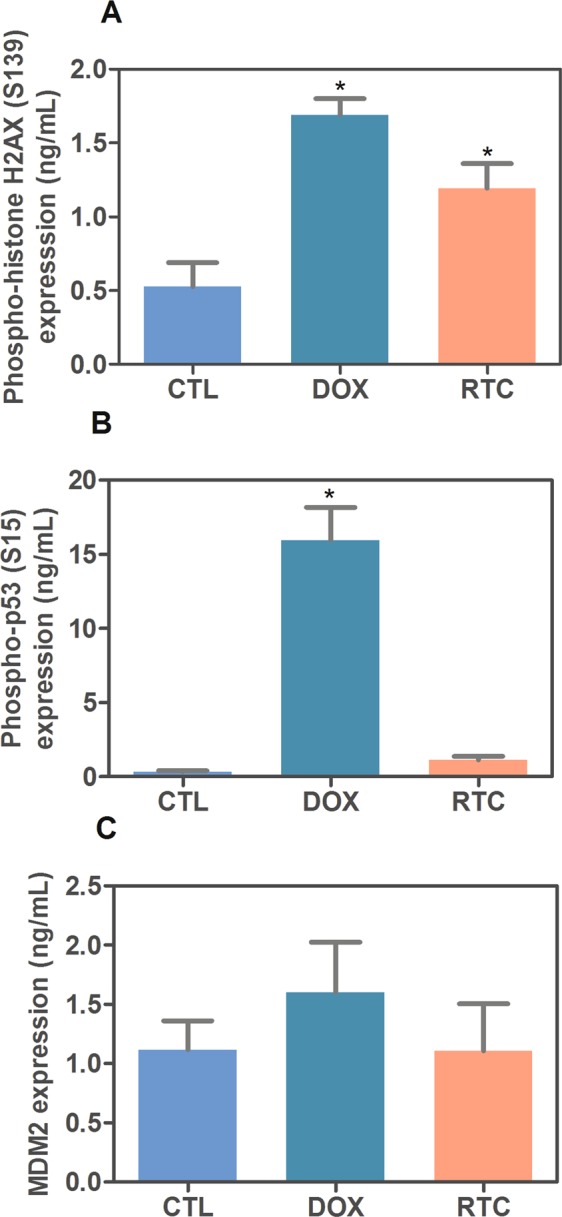
Effect of the Ru(II)-thymine complex (RTC) in phospho-histone H2AX (S139), phospho-p53 (S15) and MDM2 expressions, as determined by phospho-specific ELISA in HCT116 cells. (**A**) Quantification of phospho-histone H2AX (S139) expression. (**B**) Quantification of phospho-p53 (S15) expression. (**C**) Quantification of MDM2 expression. The cells were treated with the complex at 4 µM for 24 h. The negative control was treated with the vehicle (0.1% DMSO) used for diluting the complex, and doxorubicin (1 µM) was used as the positive control. Data are presented as the means ± S.E.M. of at the least three independent experiments performed in duplicate. *P < 0.05 compared with the negative control by ANOVA, followed by the Student-Newman-Keuls test.

**Figure 5:**
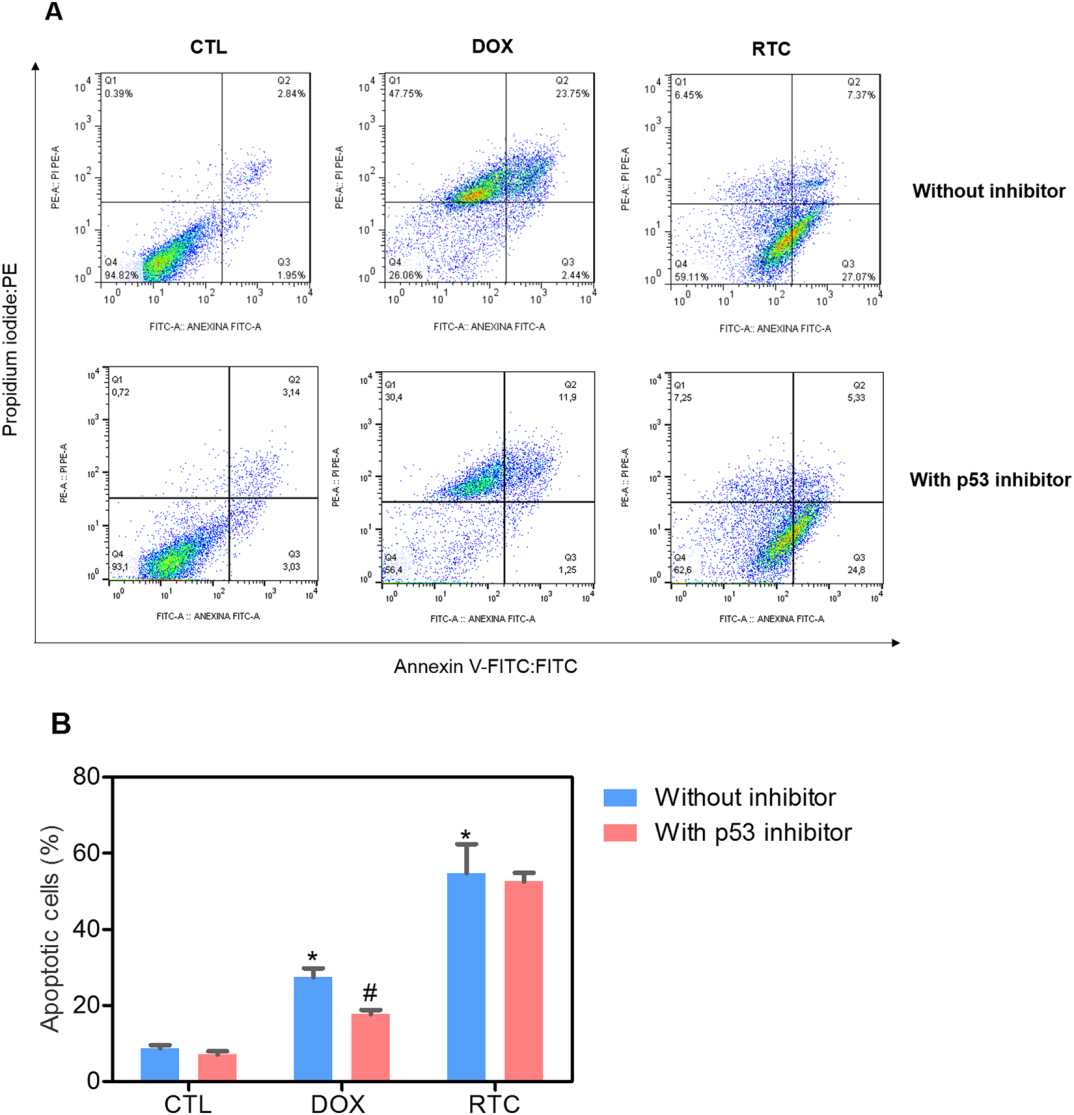
Effect of a p53 inhibitor (cyclic pifithrin-α) in the apoptosis induced by the Ru(II)-thymine complex (RTC) in HCT116 cells, as determined by flow cytometry using Annexin V-FITC/PI staining. (**A**) Representative flow cytometric dot plots showing the percentage of cells in viable (annexin V-FITC negative and PI negative cells), early apoptotic (annexin V-FITC positive, but PI negative cells), late apoptotic (annexin V-FITC positive and PI positive cells) and necrotic stages (PI positive, but annexin V-FITC negative cells). (**B**) Quantification of apoptotic HCT116 cells (annexin V-FITC positive cells). The compound cyclic pifithrin-α was used at 10 µM as p53 inhibitor inhibitor. The cells were pretreated for 2 h with the inhibitor and then co-incubated with the complex at 4 µM for more 48 h. The negative control was treated with the vehicle (0.1% DMSO) used for diluting the complex, and doxorubicin (1 µM) was used as the positive control. Data are presented as the means ± S.E.M. of at the least three independent experiments performed in duplicate. At least 1 × 10^4^ events were recorded per sample and cellular debris was omitted from the analysis. *P < 0.05 compared with the negative control by ANOVA, followed by the Student-Newman-Keuls test. ^#^*P* < 0.05 compared with the respective treatment without inhibitor by ANOVA, followed by the Student-Newman-Keuls test.

Since MAPK pathway can be activated by DNA damage and has linking with p53 signaling, we evaluated the DNA damage by quantifying the histone H2AX phosphorylation and the p53 signaling by measuring phospho-p53 (S15) (a positive regulator) and MDM2 (a negative regulator) expressions, in HCT116 cells treated with Ru(II)-thymine complex at 4 µM for 24 h. The treatment with the complex significantly increased the phospho-histone H2AX (S139) expression, but not phospho-p53 (S15) and MDM2 (Fig. 4). In addition, the co-treatment with a p53 inhibitor (cyclic pifithrin-α) did not reduce the complex-induced apoptosis in HCT116 cells (Fig. 5), indicating that DNA damage-mediated apoptotic cell death is induced by JNK/p38/ERK1/2 via a p53-independent signaling.

### Ru(II)-thymine complex inhibits HCT116 cell growth in xenograft model

**Figure 6:**
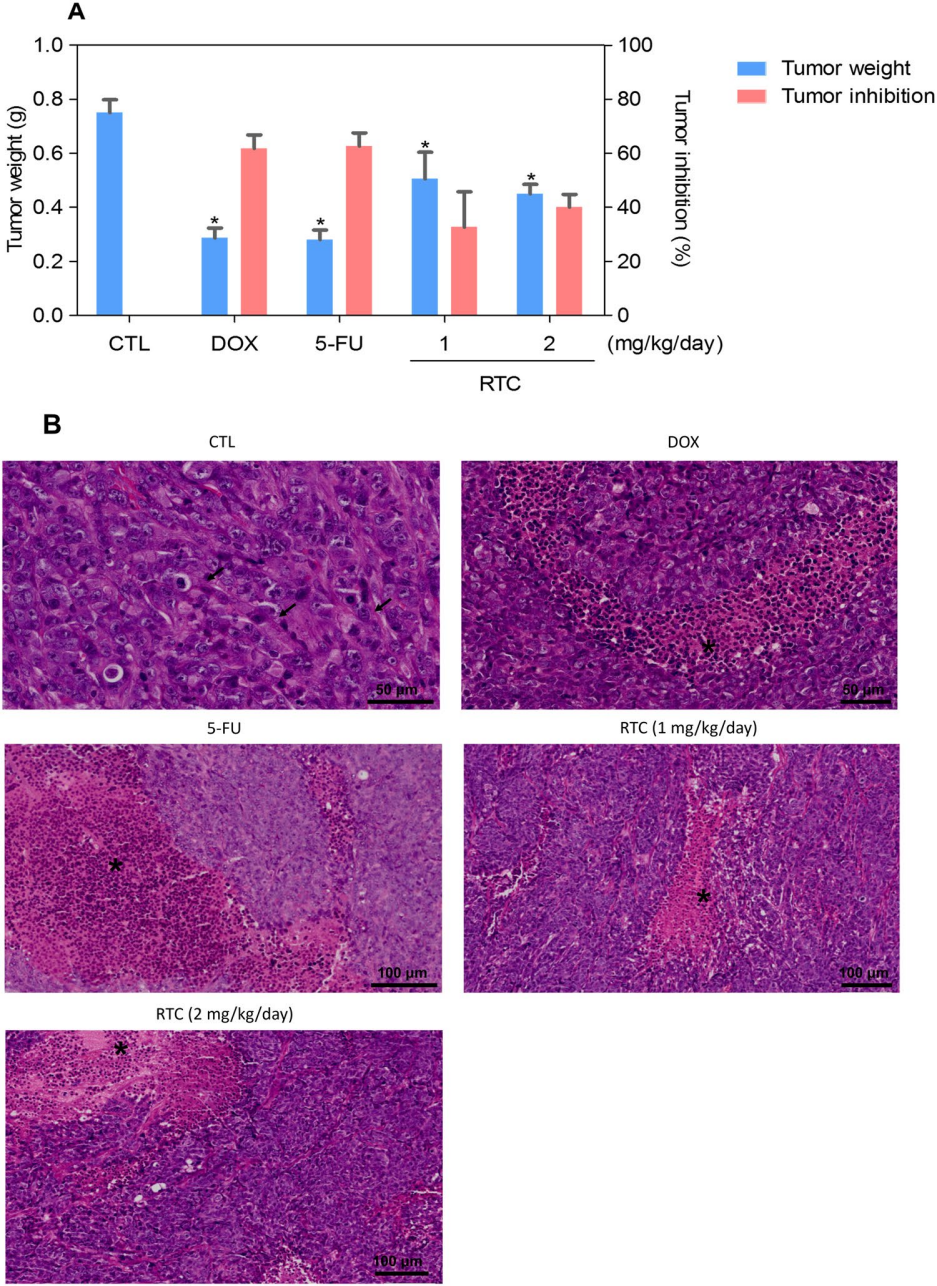
*In vivo* anti-colon cancer effect of the Ru(II)-thymine complex (RTC) in C.B-17 SCID mice with HCT116 cell xenografts. (**A**) Quantification of tumor weight (g) and tumor inhibition rates (%). Data are presented as the means ± S.E.M. of 9–23 animals. *P < 0.05 compared with the negative control by ANOVA, followed by Student-Newman-Keuls test. (**B**) Representative histological analysis of the tumors stained with hematoxylin and eosin and analyzed by optical microscopy. The asterisks represent areas with tumor necrosis and inflammation, and the arrows indicate cells in mitosis. The negative control (CTL) was treated with the vehicle (5% DMSO) used for diluting the complex, and doxorubicin (DOX, 0.8 mg/kg/day) and 5-fluorouracil (5-FU, 15 mg/kg/day) were used as positive controls.

The *in vivo* anti-colon cancer effect of Ru(II)-thymine complex was evaluated in C.B-17 SCID mice engrafted with HCT116 cells. One day after cancer cell inoculation, the animals were treated by intraperitoneal route for 15 consecutive days with the complex at dose of 1 and 2 mg/kg/day. In the end of the treatment, the mean of tumor mass weight of the negative control group was 0.75 ± 0.05 g. In animals treated with complex, the mean of tumor mass weight was 0.51 ± 0.10 and 0.45 ± 0.04 g at lower and higher dose tested, respectively (Fig. 6A). Doxorubicin, at dose of 0.8 mg/kg/day, and 5-fluorouracil, at dose of 15 mg/kg/day, were used as positive controls and showed a mean of tumor mass weights of 0.29 ± 0.04 and 0.27 ± 0.04 g, respectively. The tumor mass inhibition rate was 32.6–40.1% for the complex. Doxorubicin and 5-fluorouracil showed tumor mass inhibition rates of 61.8 and 62.7%, respectively. All tumors were classified as a poorly differentiated adenocarcinoma with a predominant solid pattern. Malignant cells exhibiting a large cytoplasm and nuclei with prominent nucleoli. Tumor-infiltrating lymphocytes were frequent in all experimental groups. In control group, mitotic figures were frequent in areas of sheets of small- to medium-sized cells. Areas of necrotic debris were more evident in the groups treated with the complex at the dose of 2 mg/kg/day and 5-fluorouracil (Fig. 6B).

Body and organ (liver, kidney, lung and heart) weights, and hematological analysis were evaluated in all groups in the end of treatment to assess the toxicological aspects. A significant reduction in the body weight of the animals treated with doxorubicin and the complex at dose of 2 mg/kg/day was observed (P < 0.05). However, no significant alterations were observed in the liver, kidney, lung or heart wet weight of any group (Table 1). Concerning the hematological parameters, only the platelets showed a significant reduction after the treatment with doxorubicin (P < 0.05). Animals treated with the complex presented hematological parameters similar to those of the negative control (Table 2).

**Table 1:** Effect of the Ru(II)-thymine complex (RTC) on body and relative organ weight from C.B-17 SCID mice engrafted with HCT116 cells.

Parameters	Non-tumor	CTL	DOX	5-FU	RTC	RTC
Dose (mg/kg/day)	—	—	0.8	15	1	2
Survival	10/10	23/23	14/22	9/10	10/10	20/20
Initial body weight (g)	22.55 ± 0.21	23.48 ± 0.23	20.10 ± 0.91	22.86 ± 0.75	22.25 ± 0.63	21.05 ± 0.41
Final body weight (g)	22.62 ± 0.38	21.98 ± 0.41	14.61 ± 0.77*	19.72 ± 0.57*	21.20 ± 0.25	18.92 ± 0.35*
Heart (g/100 g body weight)	0.52 ± 0.02	0.57 ± 0.03	0.60 ± 0.03	0.53 ± 0.04	0.56 ± 0.04	0.60 ± 0.02
Lung (g/100 g body weight)	0.8 ± 0.03	0.78 ± 0.02	0.92 ± 0.04	0.79 ± 0.06	0.77 ± 0.04	0.74 ± 0.02
Liver (g/100 g body weight)	4.52 ± 0.11	4.46 ± 0.11	4.45 ± 0.22	4.56 ± 0.25	4.91 ± 0.36	4.96 ± 0.17
Kidney (g/100 g body weight)	1.44 ± 0.03	1.50 ± 0.04	1.59 ± 0.10	1.48 ± 0.08	1.48 ± 0.09	1.61 ± 0.04

Non-tumor group represents C.B-17 SCID mice without tumor inoculation or any treatment. The negative control (CTL) was treated with the vehicle (5% DMSO) used for diluting the complex, and doxorubicin (DOX, 0.8 mg/kg/day) and 5-fluorouracil (5-FU, 15 mg/kg/day) were used as positive controls. Data are presented as the means ± S.E.M. of 9–23 animals. *P < 0.05 compared with the negative control by ANOVA, followed by the Student-Newman-Keuls Test.

**Table 2:** Effect of the Ru(II)-thymine complex (RTC) on hematological parameters of peripheral blood from C.B-17 SCID mice engrafted with HCT116 cells

Parameters	Non-tumor	CTL	DOX	5-FU	RTC	RTC
Dose (mg/kg/day)	—	—	0.3	15	1	2
Erythrocytes (10^6^ cells/μL)	8.57 ± 0.78	9.45 ± 0.51	9.12 ± 0.47	7.44 ± 0.72	8.61 ± 0.70	10.01 ± 0.37
Hematocrit (%)	45.11 ± 3.95	49.66 ± 2.90	45.61 ± 4.12	34.76 ± 1.20	38.27 ± 3.18	49.78 ± 3.03
Hemoglobin (g/dL)	12.49 ± 0.61	14.08 ± 0.27	12.28 ± 0.69	11.55 ± 0.15	11.90 ± 1.05	13.81 ± 0.57
Platelets (10^3^/mm^3^)	1084 ± 138	1082 ± 66.73	335.6 ± 28.11*	1119 ± 98.10	1341 ± 85.44	1057 ± 86.24
Mean Corpuscular Volume (fL)	54.6 ± 0.26	50.50 ± 2.5	50.86 ± 1.48	43.33 ± 0.33	43.67 ± 0,33	47.78 ± 1.19
Total leukocytes (10^3^ cells/μL)	3.09 ± 0.93	4.44 ± 0.97	2.96 ± 0.29	5.92 ± 1.86	8.20 ± 0.97	3.35 ± 0.26
**Differential leukocytes (%)**						
Granulocytes	13.92	23.04	17.89	21.35	21.36	29.65
Lymphocytes	68.68	54.31	63.11	63.20	61.96	46.18
Monocytes	14.67	26.76	17.03	15.45	17.81	24.22

Non-tumor group represents C.B-17 SCID mice without tumor inoculation or any treatment. The negative control (CTL) was treated with the vehicle (5% DMSO), and doxorubicin (DOX, 0.8 mg/kg/day) and 5-fluorouracil (5-FU, 15 mg/kg/day) were used as positive controls. Data are presented as the means ± S.E.M. of 7–14 animals. *P < 0.05 compared with the negative control by ANOVA, followed by the Student-Newman-Keuls Test.

Morphological analyses of the liver, kidneys, lungs and hearts in all groups were performed. In the livers, the acinar architecture and centrilobular vein were also preserved in all groups. Focal areas of inflammation and coagulation necrosis were observed in all experimental groups. Other findings, such as congestion and hydropic degeneration were found in all groups, ranging from mild to moderate. It is important to note that hydropic degeneration was more evident in some animals treated with doxorubicin and the complex at the dose of 1 mg/kg/day. In the lungs, the architecture of the parenchyma was partially maintained in all groups, observing a thickening of the alveolar septum with decreased airspace, ranging from focal and generalized areas in analyzed tissues. Histopathological analyses of the lungs revealed significant inflammation predominantly of mononuclear cells, edema, congestion and hemorrhage, ranging mild to severe. In the kidneys, tissue architecture was preserved in all experimental groups. Histopathological changes included vascular congestion and thickening of basal membrane of renal glomerulus with decreased urinary space were observed in all kidneys, ranging from mild to moderate. Histopathological analysis of animal hearts did not show alterations in any group.

## Discussion

Herein, we demonstrated for the first time that the Ru(II)-thymine complex causes DNA damage and apoptotic cell death in HCT116 cells mediated by JNK/p38/ERK1/2 via p53-independent signaling, and inhibits tumor cell growth in xenograft model.

MAPK signaling play a key role in the metabolic pathways of various cellular processes, including cancer cell proliferation and programmed cell death^18–20^. The classical MAPK family is composed of JNK/SAPK (isoforms JNK-1, JNK-2 and JNK-3), p38 MAPK (isoforms p38α, p38β, p38γ and p38δ) and ERK1/2, which are known to mediate many of the processes associated with growth, survival and apoptosis. We found that co-treatment with JNK/SAPK, p38 MAPK and MEK inhibitors, which inhibit the activation of ERK1/2, reduced the complex-induced apoptosis in HCT116 cells, indicating that the MAPK pathway is involved in the cell death caused by this metal complex. In addition, we confirmed that phosphorylated JNK2 (T183/Y185), p38α (T180/Y182) and ERK1 (T202/Y204) expression were augmented in the complex-treated HCT116 cells.

We found a significant increase in phospho-histone H2AX (S139) expression, indicating that this complex causes DNA damage in HCT116 cells. Numerous compounds that cause DNA damage induce apoptosis by a mechanism involving the activation of JNK and p38 MAPK, which in turn activate pro-apoptotic factors by phosphorylation^19–21^. In particular, the treatment of Ehrlich-Lettre EAC ascites carcinoma cells with the RAPTA-C ruthenium(II)-arene complex resulted in the accumulation of phospho-JNK and its substrate^22^. Chen *et al*.^23^ also showed that a ruthenium(II) complex induced JNK and p38 MAPK activation, but no ERK, in human lung carcinoma A549 cells. KP1019, which is one of the most studied ruthenium-containing compounds, has demonstrated antitumor activity against various types of cancer and has shown activation of the MAPK pathway as a mechanism of action and induction of apoptosis^24^, corroborating with our results.

The ERK pathway is initiated by mitogenic stimuli, such as growth factors, cytokines, internal metabolic stress, DNA damage, altered protein concentrations and plays an important role in the regulation of cell growth and differentiation^18,25^. It has been reported that the ERK pathway may be both oncogenic and have tumor suppressor effects, depending on the tissue-specific tumor microenvironment and the time, duration and intensity of its signal. DNA damage stimuli, such as etoposide, platinum complexes and ionizing radiation have been shown to activate the ERK1/2 pathway contributing to apoptosis^18,26–28^. In this study, we demonstrated that the Ru(II)-thymine complex causes apoptotic cell death in HCT116 cells through ERK1/2 signaling. Corroborating with our results, a ruthenium complex with xanthoxylin was previously described as able to cause ERK1/2-mediated apoptosis in HepG2 cells through a p53-independent pathway^12^.

The role of the p53 pathway was also investigated in the complex-mediated apoptosis. We observed that pre-treatment with the p53 inhibitor did not prevent the complex-induced apoptosis neither increased phospho-p53 (S15) expression in HCT116 cells, indicating that apoptosis was activated via a p53-independent pathway. The fact that p53 is inactivated in more than 50% of all cancers leads to drug resistance and therapeutic failures because some antineoplastic drugs act by a p53-dependent mechanism^29,30^. Therefore, the Ru(II)-thymine complex tested here is attractive because it induces cell death independently of p53 status. Our data corroborate with the work reported by Chow *et al*.^29^, in which Ru(II)-arene containing azopyridine, iminopyridine, chloroquine, phenanthroline and imidazole induce cytotoxicity in both wild-type p53 and p53-null HCT116 cells, indicating a p53-independent apoptosis induction by these compounds.

Regarding the Ru(II)-thymine complex antitumor effect *in vivo*, we demonstrated a significantly reduction of the tumor weight at both doses tested (1 and 2 mg/kg/day) in C.B-17 SCID mice inoculated with HCT116 cells. Other ruthenium complexes also exhibited *in vivo* antitumor activity in xenotransplantation models, such as the ruthenium(II) complex with xanthoxylin in HepG2 cells^12^, a ruthenium(II) complex with a phenylterpyridine derivative in human melanoma A375 cells^31^, a ruthenium (II) imidazole complex in A549 cells^32^ and a ruthenium(II) triazine complex against CD133 + HCT-116 (cancer stem cells derived tumor) xenografts^33^. These results indicate that ruthenium complexes, especially ruthenium (II) complexes, may have not only potent cytotoxicity *in vitro*, but also *in vivo* antitumor activity.

**Figure 7:**
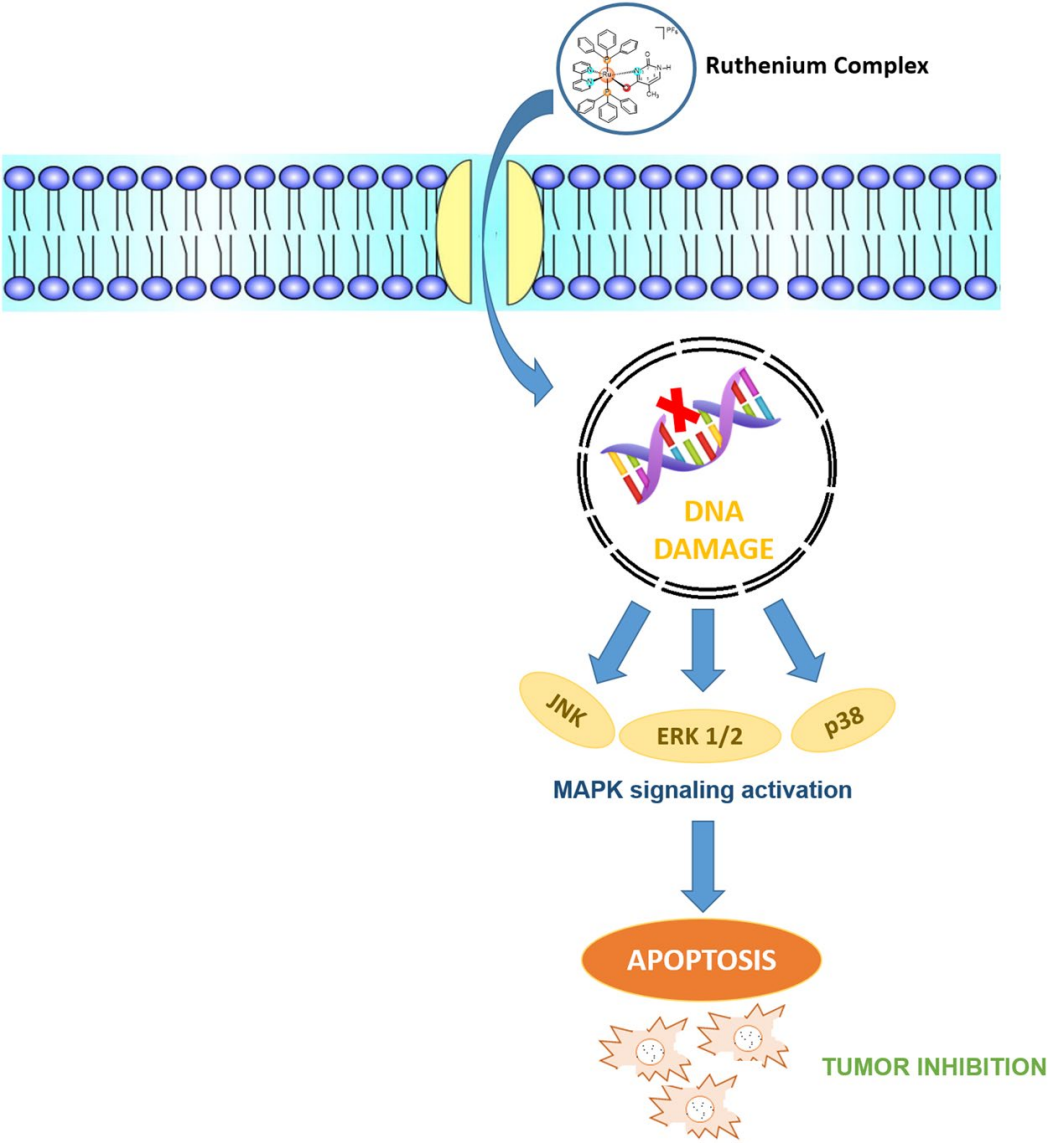
Proposal of the mechanism of action of the Ru(II)-thymine complex.

In conclusion, the Ru(II)-thymine complex causes DNA damage that triggers apoptotic cell death in HCT116 cells mediated by JNK/p38/ERK1/2 via a p53-independent signaling (Fig. 7). In addition, this molecule reduces the growth of HCT116 cells in a xenograft model, indicating that the Ru(II)-thymine complex is a promising antitumor agent and a novel anticancer drug candidate.

## Material and Methods

### Synthesis of the Ru(II)-thymine complex

The Ru(II)-thymine complex with formula [Ru(PPh_3_)_2_(Thy)(bipy)]PF_6_ was obtained at mild condition and fully characterized as previously described by Correa *et al*.^16^.

### *In vitro* assays

#### Cells

The human colon carcinoma cell line HCT116 was obtained from the American Type Culture Collection (ATCC, Manassas, VA, USA) and was cultured as recommended by ATCC. A mycoplasma stain kit (Sigma-Aldrich) was used to validate the use of cells free from contamination. Cell viability in all experiments was examined using the trypan blue exclusion assay. Over 90% of the cells were viable at the beginning of the culture.

#### Cell death detection

The FITC Annexin V Apoptosis Detection Kit I (BD Biosciences) was used for cell death detection, and the analysis was performed according to the manufacturer’s instructions. Cell fluorescence was determined by flow cytometry, and 10,000 events were recorded per sample using a flow cytometry with a BD LSRFortessa cytometer, BD FACSDiva Software (BD Biosciences) and FlowJo Software 10 (FlowJo Lcc; Ashland, OR, USA). Cellular debris were omitted from the analysis. The percentages of viable, early apoptotic, late apoptotic and necrotic cells were determined. Protection assays using a JNK/SAPK inhibitor (SP600125; Cayman Chemical), p38 MAPK inhibitor (PD169316; Cayman Chemical), MEK (mitogen-activated protein kinase kinase) inhibitor (U0126; Cayman Chemical) and p53 inhibitor (cyclic pifithrin-α; Cayman Chemical) were performed. The cells were pre-incubated for 2 h with 5 µM SP600125, 5 µM PD169316, 5 µM U0126 or 10 µM cyclic pifithrin-α, followed by incubation with the complex at 4 µM for 48 h. The concentration of the complex in HCT116 cells was previously established by Oliveira *et al*.^17^. The negative control was treated with the vehicle (0.1% DMSO) used for diluting the complex, and doxorubicin (1 µM) was used as the positive control.

#### Phospho-specific ELISA

Human phospho-JNK2 (T183/Y185), phospho-p38α (T180/Y182), phospho-ERK1 (T202/Y204), phospho-p53 (S15), total MDM2 and phospho-histone H2AX (S139) were quantified in cell lysates using sandwich ELISA kits (R&D Systems, Inc. Minneapolis, MN, USA), and the analyses were performed according to the manufacturer’s instructions. The cells were lysed in a buffer solution containing 100 mM tris, pH 7.4, 150 mM NaCl, 1 mM EGTA, 1 mM EDTA, 1% triton X-100 and 0.5% sodium deoxycholate plus phosphatase inhibitor cocktail, protease inhibitor cocktail and 1 mM PMSF immediately before use (all purchased from Sigma-Aldrich Co.). Total protein quantification was performed in each sample by Pierce Protein Assay (Thermo Fisher Scientific, Waltham, MA, USA) using BSA as standard. Absorbance at 450 nm was measured using the SpectraMax 190 Microplate Reader (Molecular Devices, Sunnyvale, CA, USA).

### ***In vivo*** assays

#### Animals

A total of 95 C.B*-*17 SCID mice (males and females, 20–25 g) were obtained and maintained at the animal facilities from Gonçalo Moniz Institute-FIOCRUZ (Salvador, Bahia, Brazil). Animals were housed in cages with free access to food and water. All animals were kept under a 12:12 h light-dark cycle (lights on at 6:00 a.m.). The animals were treated according to the ethical principles for animal experimentation of SBCAL (Brazilian Association of Laboratory Animal Science), Brazil. The Animal Ethics Committee of Gonçalo Moniz Institute-FIOCRUZ (Salvador, Bahia, Brazil) approved the experimental protocol (number 06/2015).

#### Human colon carcinoma xenograft model

HCT116 cells (2 × 10^7^ cells per 500 µL) were implanted subcutaneously into the left front armpit of the mice. At the beginning of the experiment, mice were randomly divided into five groups: group 1 animals received injections of vehicle with 5% DMSO (n = 23); group 2 animals received injections of doxorubicin (0.8 mg/kg/day, n = 22); group 3 animals received injections of 5-fluorouracil (15 mg/kg/day, n = 10); group 4 animals received injections of Ru(II)-thymine complex (1 mg/kg/day, n = 10); and group 5 animals received injections of Ru(II)-thymine complex (2 mg/kg/day, n = 20). The treatments were initiated one day after the cancer cell injection. The animals were treated intraperitoneally (200 µL per animal) once a day for 15 consecutive days. One day after the end of the treatment, the animals were anesthetized, and peripheral blood samples were collected from the brachial artery. Animals were euthanized by anesthetic overdose, and tumors were excised and weighed.

#### Toxicological aspects

Mice were weighed at the beginning and at the end of the experiment. All animals were observed for signs of abnormalities throughout the whole study. Hematological analysis was performed using the Advia 60 hematology system (Bayer, Leverkusen, Germany). Livers, kidneys, lungs and hearts were removed, weighed and examined for any signs of macroscopic lesions, color changes and/or hemorrhages. After macroscopic examination, the tumors, livers, kidneys, lungs and hearts were fixed in 4% formalin buffer and embedded in paraffin. Tissue sections were stained with hematoxylineosin staining, and a pathologist performed the histological analyses under optical microscopy.

#### Statistical analysis

Data are presented as mean ± S.E.M. Differences between experimental groups were compared using analysis of variance (ANOVA) followed by the Student–Newman–Keuls test (*p* < 0.05). All statistical analyses were performed using GraphPad Prism (Intuitive Software for Science, San Diego, CA, USA).

## Data Availability

The datasets generated during and/or analyzed during the current study are available from the corresponding author on reasonable request.
